# Towards mHealth Systems for Support of Psychotherapeutic Practice: A Qualitative Study of Researcher-Clinician Collaboration in System Design and Evaluation

**DOI:** 10.1155/2016/5151793

**Published:** 2016-03-01

**Authors:** Karin Halje, Toomas Timpka, Joakim Ekberg, Magnus Bång, Anders Fröberg, Henrik Eriksson

**Affiliations:** ^1^Young Adults Centre, Region Östergötland, 58185 Linköping, Sweden; ^2^Department of Medical and Health Sciences, Linköping University, 58183 Linköping, Sweden; ^3^Unit for Health Analysis, Centre for Healthcare Development, Region Östergötland, 58185 Linköping, Sweden; ^4^Department of Computer and Information Science, Linköping University, 58183 Linköping, Sweden

## Abstract

We examined clinicians' and researchers' experiences from participation in collaborative research on the introduction of Internet and mobile information systems (mHealth systems) in psychotherapeutic routines. The study used grounded theory methodology and was set in a collaboration that aimed to develop and evaluate mHealth support of psychotherapy provided to young people. Soundness of the central objects developed in the design phase (the collaboration contract, the trial protocol, and the system technology) was a necessary foundation for successful collaborative mHealth research; neglect of unanticipated organizational influences during the trial phase was a factor in collaboration failure. The experiences gained in this study can be used in settings where collaborative research on mHealth systems in mental health is planned.

## 1. Introduction

Various strategies have been proposed for strengthening the connection between intervention research and clinical practice in psychotherapy, for example, publication of treatment manuals supported by randomized controlled trials (RCTs) [1, 2]. Although these strategies contribute to evidence-based practice, many of them also reflect what has been called a “top-down” perspective on collecting and applying scientifically based information [3]. The widespread use of top-down methods for research-based intervention development has led to formulation of the “empirical imperialism” concept, which denotes a situation whereby researchers who themselves see few patients inform clinicians, who rarely participate in research design and therefore seldom influence what should be studied to improve psychotherapy [4]. The existence of a gap between intervention research and clinical practice is at present particularly worrisome in light of the rapid progress of Internet and mobile systems (mHealth systems) in mental health [5]. Less than two decades ago, the research-practice gap in health services meant that it could take up to 17 years before intervention research was translated into practice [6]. Although the situation is likely to have improved, the introduction of mHealth systems into psychotherapeutic practice risks being unnecessarily delayed by the research-practice gap if attention is not paid to the techniques used when these systems are adapted to clinical routines and the methods used when their effectiveness is evaluated [7].

The aim of this exploratory study is to examine clinicians' and researchers' experiences from participation in collaborative research on the introduction of an mHealth system in psychotherapeutic practice. The study is based on collection and analysis of qualitative data in accordance with the grounded theory methodology [8]. With this approach, models of social structures and processes are derived through an inductive process in which qualitative data are analysed to construct “local” theories that explain the data in the context in which they were gathered. The context where data were collected for the present study is a research collaboration aimed at developing and evaluating mHealth support of cognitive behavioural therapy (CBT) for young people with mental health problems.

*Research Setting*. In 2008, a research collaboration was initiated between the Young Adults Centre in Linköping, Sweden, and researchers at Linköping University. The Young Adults Centre provides psychological services to young adults (16–25 years of age) in Linköping, Åtvidaberg, and Kinda municipalities (combined population 145,000) in Östergötland County, Sweden [9]. The intervention programme follows a predefined structure with a basis in CBT, individually customized into one of four threads based on the client's mental health problems: (1) anxiety, (2) depression, (3) anxiety and depression combined, or (4) decreased well-being without specific anxiety or depression. It consists of six sessions, each lasting 45 minutes. Personalized homework tasks are also assigned to be completed before the next session. A report on the client's progress, including whether the client has expressed any suicidal intent, is routinely recorded. Clients receive whatever psychological treatment their therapist prescribes in parallel with other ongoing interventions, such as provision of practical or social assistance at the employment office, and further medical investigations. Clients continue to receive pharmacotherapy if previously prescribed by their physician. At the last session, a personalized postintervention knowledge and skills maintenance programme is outlined for each client to be used after discharge from the centre. The centre employs four psychotherapists with training in CBT. Self-referral by telephone is the only means for clients to contact the centre. Preliminary admission to the service is regulated by therapist triage by telephone. At the first session in the centre, the nature of the client's problems is evaluated. After this evaluation, clients are offered one of two alternatives: self-help instructions within the remaining session for clients with minor problems or a structured psychological intervention programme.

The aim of the research collaboration was to develop and evaluate mHealth support of CBT. The collaboration involved two research steps: meetings and workshops where the mHealth systems and trial design were conceived and the performance of the trial. The clinical trial was assessed and approved by the regional ethical review board in Linköping (Dnr. 111-09). For the first step, a PD approach was used whereby therapists, researchers, and information system developers worked together. The mHealth system was outlined in a series of design meetings with therapists and technically implemented as a smartphone application. The evaluation of the mHealth system was performed as an RCT [10]. The main aim of the trial was to examine whether mHealth systems can lead to improved treatment. A secondary goal was to study whether the mHealth technology aided treatment adherence. The inclusion criteria for the trial was that the client was 18–25 years of age and had scored seven points or higher on the anxiety subscale of the Hospital Anxiety Depression Scale (HADS) [11].

*Background*. A first basis for efficacious introduction of mHealth systems in psychotherapy is a reciprocal knowledge transfer between information system designers and clinical practitioners. Several approaches for human-centred information systems development have been reported, ranging from participatory design (PD) [12] to usability engineering [13] and contextual design [14]. PD was introduced for use in situations when social factors and work practice experiences were anticipated to be of particular importance for successful system implementation [15]. The first generation of PD methods, also denoted by collective systems design [16], was developed for industrial workplaces with support from trade unions, with the objective that the resulting IT-supported production systems should enhance workplace democracy and increase worker autonomy, skill set, and task variety. System users were given direct influence on the design through participation in design groups where they contributed with organizational and work task knowledge. The design methodology, based on techniques such as mock-up evaluations and future workshops [17], was straightforward to learn and put low demand on users' prior knowledge. A second generation of PD was characterized by a shift towards the commercial setting and by embracing teamwork and finding points of contact with the area of computer-supported cooperative work [18, 19]. However, several authors pointed out that even this generation of PD was seldom used in large, product-oriented projects and that, once it was applied, it resulted in only small-scale stand-alone IT applications [20, 21]. A third generation of PD emerged as a response to this criticism, providing means for adaptations to organizational trends, large organizations and projects, interorganizational collaboration, and networking, and with increased consideration of third parties (clients, customers) in the systems development process [22]. In parallel, design methods with their origin in PD have also been incorporated into general methods for the development of eHealth systems including those based on ubiquitous technologies [23].

Another basis for the introduction of mHealth systems in routine psychotherapy is a functional collaboration between clinical researchers and practicing psychotherapists [24]. For several decades, efforts have been made to reduce the research-practice gap in psychotherapy. An early attempt was presented in the Boulder model, a structure for scientist-practitioner collaboration that was developed in the 1950s [25]. However, before long, studies on psychological treatment in mental health practice showed that when the selective inclusion criteria used in experimental settings could no longer be applied, the observed effects on patients decreased [26]. Although a strong consensus that a scientific approach should permeate psychotherapeutic practices remains, it has repeatedly been suggested that experimental research can lead to unrealistic and overly optimistic beliefs about what evidence-based treatment can provide in everyday clinical practice [27]. Inspired by the work of Sobell [28], the Society of Clinical Psychology, Division 12 of the American Psychological Association (APA), began therefore in 2010 an initiative to build a two-way bridge between research and practice [29]. A feedback mechanism was presented similar to that employed by the FDA; that is, after the introduction of an evidence-based treatment, practitioners can offer feedback based on their clinical experiences in using the treatment in practice. This two-way bridge model for obtaining feedback on the use of empirically supported therapies in practice was expanded in 2011 to become a collaborative effort together with the Division of Psychotherapy of the APA—Division 29. The practice research network (PRN) is another recent approach to facilitate integration between research and practice. A PRN is based on active collaboration between researchers and clinicians in the development of relevant clinical studies that are also profoundly scientific [1, 3]. This approach suggests that collaboration can help the psychotherapy field move beyond the efforts of building bridges between research and practice. Rather than conceiving the scientist-practitioner collaborations as links between two groups of individuals, creation of unified landscapes of knowledge is conceived where clinicians and therapists are working together on clinically actionable and scientifically rigorous studies. A concept closely related to the PRN approach is psychotherapy integration [30]. This notion highlights structural advances with regard to theoretical integration, technical eclectic, common factors, and assimilative integration and has been suggested for psychotherapy research and practice in areas such as harmful effects, therapist effects, practice-oriented research, and training.

## 2. Methods

The study was based on a cross-sectional design and used qualitative methods for data collection and analyses. According to Swedish legislation, the research did not require a separate review of ethical considerations.

### 2.1. Data Collection

Semistructured interviews took place with four psychotherapists and five researchers who had participated in the collaborative research at the Young Adults Centre. The interviews were conducted by a behavioural scientist who had not participated in the research study. The interview questions were aimed at uncovering what the clinicians and researchers had experienced when participating in the multidisciplinary research, as well as their recommendations for future studies [3]:

- What have you found to be most interesting and/or useful in your participation in the study?
- What have you found to be difficult and/or frustrating with your participation?
- What, if any, parts of this study have benefited and/or have entailed a risk to patients?
- What has been the most frequent and/or important obstacle to conduct the study? and
- If you confronted significant barriers when the study was conducted, what, if anything, has helped you to deal with these obstacles?

Further clarifying questions were posed when found necessary. The interviews lasted about 30 minutes and were transcribed from audio recordings. The transcribed data comprised about 30 printed pages of text.

### 2.2. Data Analysis

The data analysis based in the grounded theory methodology involved dividing the interview data into smaller units, conceptualization and coding of these units, and combining the labelled units into increasingly complex structures [8]. Initially, interview data were read thoroughly and notes were taken. At each step of the analysis, the data from two groups (researchers and clinicians) were analysed both separately and combined in order to allow comparisons. Three coding steps were performed: open coding, axial coding, and selective coding. These coding steps were performed iteratively, as they were revisited when categories or categorizations needed to be adjusted or added to. The open coding involved breaking down the interview data into smaller units, and iteratively examining, conceptualizing, comparing, and categorizing these units. Examples of typical codes applied at this level include “attitude-cooperation-researcher” and “experience-planning-clinician.” In the axial coding, the categories of data units were related to each other by searching for associations between them. These associations were sought on the basis of, for instance, consequences, context, interactions, and influencing factors. At this level, the categories involved instances such as “cooperation endpoints,” “surprising events,” and “inspiring happenings.” In the selective coding, the main (central) categories were selected and how they related to each other was tentatively described. For this categorization, the categories were divided into those associated with objects manufactured in the research process and social phenomena, respectively. Thereafter, the previous coding steps were revisited in order to identify categories that needed to be refined or added. Finally, a small-scale theory was formulated in terms of graphical models based on a conditional matrix, that is, charts that suggest causality and interactions between the different artefacts and phenomena identified in the analyses. The analyses were performed by the first author (Karin Halje). Preliminary results were then presented to the remaining authors. Adjustments were then made based on the feedback obtained. The procedure was repeated until no further significant comments emerged.

## 3. Results

Three manufactured objects and three social phenomena constituted the central concepts in the clinicians' and researchers' accounts of their experiences from the two phases of the collaborative research process (Table 1).

### 3.1. Participatory Design Phase

**Figure 1:**
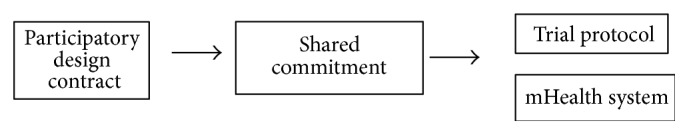
Model of the relationships identified in the analysis of the participatory design phase.

At the beginning of the PD phase, the researchers and the clinical system users jointly developed a structure for the collaborative research process. This structure was documented in the “participatory design contract.” This contract defined the structure and process of this phase and included descriptions of, for instance, the frequency, length, content, and leadership of the joint meetings that constituted the research process (Figure 1). One researcher expressed “It is important to have joint meetings at the beginning so that you really share what it's all about. And then go through the routines even more clearly” (Researcher 2). At these initial meetings, a shared foundation for the research was developed, including a mutual interest in each other's (researchers and clinicians) activities. This shared commitment to the foundations also included an openness to learn from each other, as expressed in the following quotes: “It has been interesting to have had the opportunity to understand how to plan research, because I have not been involved in such undertakings before” (Clinician 4). “… The most rewarding was the cooperation between the practitioners, technical researchers, and clinical researchers. I think this was interesting and fun” (Researcher 3). An interest in each other's work practices was noted among the clinicians, which provided them with motivation for increased involvement in the study. Another clinician expressed “… we could be more consistent in how we actually work” (Clinician 2). In this collaborative setting, the mHealth system and the trial protocol were elaborated. Implementation of the system based on the clinicians' preferences further increased the clinicians' commitment to the research. One of the clinicians stated that “It was interesting to be involved in designing an app that may help people cope with difficult situations when anxiety problems are encountered” (Clinician 4). Only a few negative experiences were reported from the PD phase. An example was when participating in the PD procedures interfered with and influenced the therapeutic process. A clinician explained that “… it was [sometimes] hard to decide if I really should squeeze in participation [in the PD process], because clients may have come with difficult problems and I could have been way behind my schedule already” (Clinician 4).

### 3.2. Trial Phase

**Figure 2:**
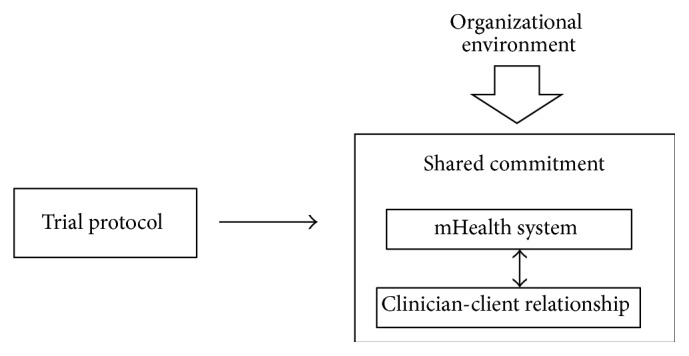
Model of the relationships identified in the analysis of the trial phase.

In the trial phase of the collaborative research, organizational structures, systems, and processes that were not considered and controlled in the trial protocol were found to influence the research setting, and, in consequence, the shared foundations and commitment (Figure 2). At the start of this phase, the trial protocol that was jointly defined in the PD phase was approved by the regional research ethics committee and recorded in an international clinical trial registry (NCT01205191 at clinicaltrials.gov). In consequence, it was no longer possible to make substantial changes to the research process. However, a number of issues not addressed in the protocol were found to affect this process. One example of such external influence is that the legislation, procurement regulations, and bureaucracy associated with the payment of mobile network operator charges at times made the mHealth system unusable even though it was technically functional. One of the researchers explained that these influences were not easy to foresee: “I should have made sure I had enough research budget so I could solve these everyday problems without having to go through a lot of individuals to obtain permission to complete the payments and ensure that there was money in the account” (Researcher 3). Also, when they were encountered and comprehended as facts, problems with their origin outside the clinical and academic contexts in which the research was planned were not easy to solve: “… these mobile phone call payments were one of those things that caused problems. The regulations and procurement procedures made me to try to improvise a solution [outside the university routines] that worked with the actual network contracts” (Researcher 8). Another issue had its basis in the fact that the study was carried out at a small clinical unit with only four full-time positions. This situation meant that the trial was noticeably vulnerable to spells of absence in the clinician group. Throughout the study period, at least one of the regular staff members was on parental or sick leave. In parallel, three of the four regular clinicians were also absent part-time (30% each) for academic studies. Although replacements were employed, the unit was not adequately staffed at times in relation to client pressure. These problems were expressed by one of the researchers as follows: “… the clinic reflects working life of today. Employees are on sick leave or parental leave. New employees arrive and others disappear for good. These are not the kind of conditions you want to face when you are going to conduct a scientific evaluation, but the fact is reality. … This turnover … needs to be taken into account when [a trial] is organized. And also who is holding the responsibility for what should be decided” (Researcher 6). The replacements who were introduced into the trial received information individually about the study protocol by a member of the regular staff. Although each temporary staff member received individual instructions, their knowledge and commitment to the study were not at the same level as those of the regular staff. A clinician commented as follows: “Another thing that has been difficult and frustrating is the lack of continuity in the clinical staff presence …, which has made it necessary to hire replacements …. This has had an impact on the trial” (Clinician 5).

Both the clinicians and the researchers perceived that organizational and bureaucratic factors with their origin outside the clinical and academic contexts in which the research was planned had come to influence the process and outcome of the clinical trial. However, although the clinicians believed that the unforeseen issues with the mHealth system, such as the problems with the smartphone payments, affected the treatment quality negatively, they also found that the system affected the treatment positively when it worked and brought a new dimension to the clinical practice. This stance with multiple perspectives is exemplified by the following statements: “I think that we have provided a better service via the app due to the fact that young people can use it to finish their homework assignments more easily than on paper. And they can more easily retrieve information to read about their problems …. [The system] also makes it easier for me to read about the homework I give them” (Clinician 4). The different organizational influences on the trial process thus made it hard for the clinicians themselves to recognize their original treatment-as-usual setting and whether the different experimental conditions involving the mHealth system had significantly improved or distorted clinical practice.

## 4. Discussion

The aim of this study was to explore clinicians' and researchers' experiences from participating in collaborative research on mHealth systems for support of psychotherapeutic practice. We identified objects manufactured in the research process (the PD contract, the trial protocol, and the mHealth technology) that constituted the reference points for the participants' experiences and observed consensus that, in the PD phase, mutual learning in regular meetings, shared commitment to the multidisciplinary design group, and daily communication between researchers and clinicians characterized the collaborative research. However, in the trial phase, organizational factors with their origin outside the setting in which the study was planned were found to have had a decisive influence on the experiences. In addition, in this phase, the researchers' and clinicians' experiences differed; the clinicians provided several post hoc examples of negative influences on clients from study participation, whereas the researchers explained that it was difficult to foresee such side effects in detail and they regarded these effects as a part of the knowledge obtained. Differences between researchers and clinicians have previously been explained by the fundamentals of their respective professions [31]; researchers strive for maintenance of controlled research conditions and clinicians focus on the best therapy for the client. These explanations are thus applicable to the present study. Nevertheless, despite these foundational differences, a shared perception of the collaborative research was created and sustained in the PD phase of the research by meetings where each other's “cultures/languages” were learnt and knowledge about each other's areas of activity was developed. Establishing interpersonal relationships, trust, and a shared culture/language has also been reported from other settings as essential factors for building sustainable collaborations between psychotherapists and researchers [32]. Yet, the prestudy differences in professional fundamentals became apparent again in the evaluation phase, when, according to the trial protocol, the clinicians were left to solve the problems that occurred independently. In consequence, as the technical-practical problems accumulated, the trial protocol was considered by the clinicians to interfere with the psychotherapy, and, in particular, introduced an adverse impact on creating alliances with clients. To minimize these effects, we infer that the ways that the evaluation procedures could affect clients should have been discussed in even greater detail in the design phase.

The results show that the PD approach seemingly was functional for the development of mHealth systems to support psychotherapeutic practice; obstacles to the collaborative research mainly surfaced in the trial phase. In the trial phase, uncontrolled influences from external social systems, such as legalization and staff management routines in the health service organization, had an impact on the trial performance, dedication to the collaborative research and, as perceived by the clinicians, the psychotherapeutic treatment. Thus it is important to identify and control possible external influences at the design phase in collaborations aiming to develop mHealth systems that support psychotherapeutic practice. The problems that arose with the smartphones in the present study can be seen as evidence of the need to ascertain that the planned mHealth system can be safely sustained over sufficient periods of time in the evaluation setting before the formal trial is initiated. In the present study, a pilot test involving three young adults, who not were clients, was performed over a 2- to 3-week period. This test was deemed successful and it was concluded that the trial could begin. What was not taken into account was that, in the RCT, smartphone use by one client could well extend to several months (which was a normal treatment time). This led to a need to introduce a way for a third party (the researchers) to pay for the clients' smartphone charges in the clinical setting over lengthy periods of time, yet not allowing abuse of the smartphone accounts. It is likely that if the pilot tests had been performed in a setting that more closely resembled the RCT conditions, including real clients over a longer period of time, these problems would have been detected and solved before the trial was initiated. This said, it is also probable that involvement of clients already in the PD phase of the collaborative research would have prevented at least some of the problems associated with phone use in practice. These experiences can be added to the recently published guidelines for evaluations of mobile mental health applications [33].

The present study has both strengths and limitations that need to be considered when interpreting the results. In the qualitative data analysis context, theoretical sensitivity means that professional experiences and personal life understandings can be utilized in data processing [8]. Theoretical sensitivity thus denotes an extended ability to recognize important data and meaningful interpretations in a particular context. A strength of the present study is that the theoretical sensitivity of the multiprofessional group analysing the data can be regarded as having covered all of the most important aspects of the study setting, that is, psychotherapeutic practice as well as clinical and informatics research. An associated potential limitation of the study is that the research was performed in a self-evaluation setting; all authors also participated in the collaborative research. Holme and Krohn Solvang [34] mentioned four elements that are crucial for avoiding bias when planning interviews and interpreting the collected data: (a) the interview themes (topics may be sensitive to discuss), (b) respondent roles (expectations on “correct” behaviour), (c) respondent competences (knowledge of interview topics), and (d) interview setting (the environment restricts data provision). To decrease the risk of interview topics being perceived as too sensitive to discuss, in this study, a behavioural scientist who did not participate in the collaborative research was appointed to conduct the interviews. Also, the behavioural scientist was asked to adjust each interview setting according to the respondent's requests. Five researchers, one technician, and four clinicians were interviewed, all of whom had participated in the research study from the beginning and should therefore have had sufficient competence to provide reliable data. Due to their lesser knowledge of the research process, the two replacements who participated for short periods of time were not interviewed; the administrative head of the Young Adults Clinic was also not interviewed. However, during the data analyses, it was found that interviews with these clinicians would probably have added further information. It is difficult to assess whether role behaviours influenced the results. The data provided in the interviews and the analysis may have been biased by prearranged positive or negative attitudes to the project or intentions to “save face” or point out scapegoats. However, during the initial analysis, the first author was careful not to let her own interests influence the process by constantly writing down notes and memos [8]. This process was repeated when the remaining authors iteratively reviewed the preliminary results and provided comments. Theoretical thoughts appearing during the course of the analysis were thus documented when moving from smaller units of analysis to broader categorization to ensure that the analysis was based on trustworthy and nonpartial interpretations of the data. We therefore trust that role behaviours had a minute or no influence on the results presented.

## 5. Conclusions

This study of clinicians' and researchers' experiences from participating in collaborative research on the introduction of an mHealth system in psychotherapeutic practice showed that the application of PD techniques narrowed the gap between researchers and clinicians in the initial system design phase, whereas maintenance of the collaboration during the trial phase was disturbed by unforeseen organizational and bureaucratic problems. The main lessons learnt are that the correctness and soundness of the central objects developed in the initial design phase (collaboration contract, trial protocol, and system components) constitute the necessary foundation for successful collaborative research; lack of preparedness for influences from unanticipated social influences during the trial phase is a factor in collaboration failure. Before the evaluative trial, the functions of the mHealth system and details of the trial protocol should be verified by extended pilot tests involving real clients in order to avoid negative influence on the therapist-client relationship, and, correspondingly, on the clinicians' commitment to participate in the research. The experiences gained in this study can be used in other settings in which collaborative research on mHealth systems in psychotherapeutic practice is planned.

## Figures and Tables

**Table 1:** Central concepts in the participants' accounts of their experiences from the collaborative research.

Manufactured objects	
A1	Participatory design contract: the document describing the mHealth system development process; that is, how the joint meetings were to be led, the frequency of meetings, the communications routines, and so forth
A2	Trial protocol: the document describing the procedures that were to be followed before and after a client had agreed to be included in the evaluation trial
A3	mHealth system technology: the software applications in smartphones and on the server, the clinicians' interface, and the data storage and communication infrastructure
Social phenomena	
SP1	Shared commitment: motivation and interest among the parties involved in the research
SP2	Organizational environment of the mHealth system: legislation, bureaucratic formalities, staff replacements, workload, and so forth
SP3	The clinician-client relationship: the relationship between the clinician and client during the treatment process and its quality with regard to factors such as alliance, adherence, homework completion, and so forth
